# Global, regional, and national burden of acute glomerulonephritis from 1990 to 2021 and future trend predictions until 2036: a systematic analysis using the Global Burden of Disease Study 2021

**DOI:** 10.3389/fpubh.2025.1593055

**Published:** 2025-07-16

**Authors:** Yangtian Jiao, Xing Chen, Tianyu Zhang, Changyu Ma, Congrong Shen, Bo Yu

**Affiliations:** ^1^Department of Urology, China–Japan Friendship Hospital, Beijing, China; ^2^China–Japan Friendship Hospital, Beijing, China; ^3^Peking Union Medical College and Chinese Academy of Medical Sciences, Beijing, China; ^4^Division of Cardiology, Department of Internal Medicine, Tongji Hospital, Tongji Medical College, Huazhong University of Science and Technology, Wuhan, China; ^5^Hubei Key Laboratory of Genetics and Molecular Mechanism of Cardiological Disorders, Wuhan, China

**Keywords:** acute glomerulonephritis, Global Burden of Diseases, incidence, prevalence, disability-adjusted life years

## Abstract

**Objective:**

This study aimed to analyze the global, regional, and national burden of acute glomerulonephritis (AGN) from 1990 to 2021 and project its trends through 2036 using data from the Global Burden of Disease (GBD) study.

**Methods:**

Using the GBD 2021 dataset, we evaluated the burden of AGN in terms of incidence, mortality, and disability-adjusted life years (DALYs). Age-standardized rates (ASRs) were calculated, and trends were assessed using the Estimated Annual Percentage Change (EAPC) measure. Decomposition analysis quantified the contributions of population growth, aging, and epidemiological changes. Projections for AGN burden were modeled using the Auto-Regressive Integrated Moving Average (ARIMA) approach.

**Results:**

From 1990 to 2021, the global age-standardized incidence rate (ASIR) of AGN declined by 46.09%, and the age-standardized DALY rate decreased by 65.87%. Middle-Socio-Demographic Index (SDI) regions exhibited the highest burden, while high-SDI regions showed increasing trends in mortality and DALY, potentially linked to autoimmune and systemic conditions. Decomposition analysis highlighted the role of epidemiological improvements, balanced by the impacts of population growth and aging. Projections indicate a continued global decline, with a 43.81% reduction in the ASIR and a 62.92% decline in the age-standardized DALY rate by 2036.

**Conclusion:**

Despite significant global progress, disparities persist, particularly in low- and middle-income regions. Targeted interventions, enhanced diagnostic capabilities, and strategies that address socioeconomic determinants are essential to achieving a equitable reduction in the AGN burden. This study emphasizes the importance of global and regional strategies in improving AGN outcomes worldwide.

## 1 Introduction

Acute glomerulonephritis (AGN) is a significant global health concern characterized by the rapid onset of glomerular inflammation, which can result in hematuria, proteinuria, hypertension, and, in severe cases, acute kidney failure (1–4). The etiology of AGN is diverse, encompassing post-infectious causes, such as streptococcal infections, autoimmune disorders, and systemic diseases. It is not only a common cause of acute kidney injury (AKI) but also a significant contributor to chronic kidney disease and end-stage renal disease (ESRD), imposing long-term healthcare challenges to patients and healthcare systems (CKD) (5–7). Moreover, the interaction between AGN and metabolic syndromes (e.g., diabetes and hypertension) is becoming increasingly evident. It is reported that a high body mass index (BMI) and diabetes can exacerbate kidney damage in AGN patients, while the inflammatory response triggered by AGN may accelerate the progression of cardiovascular-kidney-metabolic (CKM) syndrome (6). Historically, AGN has been associated with substantial morbidity and mortality, particularly in regions with limited healthcare resources and high prevalence of infectious diseases (8–10). Advances in medical care, improved public health infrastructure, and targeted interventions have likely influenced the global epidemiology of AGN, but disparities persist due to variations in socioeconomic development, healthcare access, and regional disease dynamics (11).

The Global Burden of Disease (GBD) study provides a comprehensive framework for evaluating diseases, injuries, and risk factors across 204 countries and territories. Using a systematic approach, the GBD study offers insights into the incidence, mortality, prevalence, and disability-adjusted life years (DALYs) of various conditions, enabling both temporal and spatial comparisons (12–14). While GBD analyses have extensively documented trends in diseases such as CKD and cardiovascular conditions, AGN has received comparatively less attention (15–17). Previous epidemiological studies have provided foundational insights into the burden of AGN. Notably, Guo et al. analyzed Global Burden of Disease (GBD) data from 1990 to 2019, reporting a global decline in age-standardized incidence (−1.84% annually) and mortality rates (−2.89% annually), with persistent disparities in low- and middle-income countries. Their study identified East Asia and South Asia as high-burden regions, driven by post-infectious AGN in children and aging-related cases in adults (4). Regions with lower SDI values often experience disproportionately higher burdens due to factors such as inadequate sanitation, limited access to healthcare, and a higher prevalence of infectious diseases (4). Examining how these sociodemographic factors influence AGN is critical for identifying high-risk populations and tailoring effective interventions.

The global burden of AGN has likely been shaped by demographic transitions, shifts in infectious disease patterns, and advances in medical care (18). The burden of acute glomerulonephritis (AGN) is particularly pronounced in resource-limited regions. It is reported that 51% of children with AGN come from socioeconomically disadvantaged households in Nepal, and the incidence of AGN is significantly higher in rural areas compared to urban centers due to poor sanitation and high rates of streptococcal infections (19). These regions commonly face challenges such as inadequate diagnostic capacity (e.g., limited access to serum complement testing), delayed treatment (e.g., shortage in antibiotics and dialysis resources), and a lack of long-term follow-up, which collectively increase the risk of AGN progressing to chronic kidney disease (CKD). Moreover, the high hospitalization rates associated with AGN and the resulting loss of productivity (as children and young adults constitute the majority of affected individuals) further exacerbate the financial strain on families, creating a vicious cycle of “disease and poverty” (20).

This study aims to provide a comprehensive analysis of the global, regional, and national burden of AGN from 1990 to 2021 using GBD data. By examining temporal trends in incidence, mortality, and DALYs, as well as variations by age, sex, and sociodemographic development, this study seeks to fill critical gaps in understanding the epidemiology of AGN. Future projections of AGN burden will offer valuable insights for public health strategies and healthcare resource allocation. This study aims to inform evidence-based interventions that reduce the burden of AGN and improve global health outcomes.

## 2 Methods

### 2.1 The GBD study

The GBD study combines extensive data sources to generate comprehensive estimates of health outcomes, injuries, and risk factors across different geographic and temporal scales. The 2021 iteration of the GBD study evaluates mortality, incidence, prevalence, years of life lost (YLLs), years lived with disability (YLDs), and DALYs for 371 diseases and injuries, 288 causes of death, and 88 risk factors across 204 countries and territories. The methods used for data collection and analysis have been rigorously documented in previous reports. To ensure comparability across populations, all rates are standardized to the GBD world population and expressed per 100,000 individuals. Uncertainty intervals (95% UIs) are provided for all estimates to reflect potential biases, measurement limitations, and uncertainties in the modeling process. This study adheres to the Guidelines for Accurate and Transparent Health Estimates Reporting (GATHER), ensuring transparency and reproducibility.

### 2.2 Estimation of the burden of AGN

The GBD 2021 study employed sophisticated modeling approaches to estimate the burden of AGN. AGN was identified using the International Classification of Diseases and Injuries (ICD code for AGN: N00-N01.9 for ICD-10 and 580-580.9 for ICD-9). Disease incidence and prevalence were derived using DisMod-MR 2.1, a Bayesian meta-regression tool that integrates diverse disease parameters, epidemiological patterns, and geospatial data to generate reliable estimates (21, 22). Mortality estimates were obtained using the Cause of Death Ensemble Modeling (CODEm) framework, which analyzes vital registration and verbal autopsy data, including data with non-specific coding, and applies rigorous adjustments to enhance accuracy (21, 22). By incorporating multiple models, CODEm improves the precision of mortality estimates. These methodologies were applied to the 2021 dataset to produce a comprehensive assessment of AGN burden. This integrated approach accounts for variations in study design and data quality across sources, ensuring consistency and robustness in estimates of incidence, prevalence, and mortality. The disease burden, expressed in DALYs, was calculated as the sum of two components: YLD, reflecting the impact of living with the condition, and YLL, capturing the toll of premature mortality.

### 2.3 Statistical analysis

To analyze trends in age-standardized rates (ASRs) for AGN incidence, mortality, DALYs, and prevalence, this study utilized the Estimated Annual Percentage Change (EAPC) metric. ASRs were calculated per 100,000 population using a weighted formula, where age-specific rates (*a*_*i*_) were adjusted by the corresponding population weights (ω) of the standard population across all age groups (*A*): $ASR=\frac{\overset{A}{\underset{i=1}{\sum }}a_{i}\omega_{i}}{\overset{A}{\underset{i=1}{\sum }}\omega_{i}}\times 100,000$. EAPC was determined through a regression model that examined the logarithmic trend of ASRs over time. The model, expressed as *Y* = α + β*X* + *e*, defines *Y* as the natural logarithm of the ASR, *X* as the calendar year, α as the intercept, β as the slope (representing the annual rate of change), and e as the error term. The EAPC was then calculated as 100 × [exp(β) – 1], with 95% confidence intervals (CIs) provided to assess precision. A positive EAPC with a lower CI bound above zero indicated an increasing trend, while a negative EAPC with an upper CI bound below zero signaled a decreasing trend. If neither condition was met, ASRs were considered stable. To explore the relationship between AGN ASRs and the Socio-Demographic Index (SDI), Spearman's rank correlation was employed. Additionally, future projections of the AGN burden were modeled using an Auto-Regressive Integrated Moving Average (ARIMA) approach (23). Long-term projections can be uncertain because they assume past patterns will continue, but future changes, such as new treatments or policies, might disrupt this. Multiple approaches were used to address these potential uncertainties. Specifically, 95% confidence intervals were used to quantify statistical uncertainty. Robust historical data from GBD 2021 were used to reduce input uncertainty, and the comparison of predicted trends with historical trends was conducted to validate the results. All data analyses and visualizations were conducted using the World Health Organization's Health Equity Assessment Toolkit and R statistical software (version 4.4.0), ensuring accurate computation and clear graphical representation of findings.

### 2.4 Decomposition analysis

This study utilized decomposition analysis to identify and quantify the key drivers behind changes in the burden of AGN. Aging, population growth, and epidemiological changes are key factors in analyzing disease burden, as they capture shifts in demographic and health outcomes. Aging reflects the impact of an aging population on disease risk, population growth accounts for the increased number of at-risk individuals, and epidemiological changes capture the effects of health system improvements. Thus, these factors are standard in GBD studies, ensuring that the analysis is consistent with global health research practices and provides policymakers with actionable insights. Decomposition analysis separates the effects of population growth, aging, and epidemiological shifts, enabling an assessment of their contributions while accounting for other variables (24, 25). The analysis specifically targeted changes in the ASIR, ASDR, and age-standardized DALY rate for AGN. The relative impact of each factor on overall changes in ASRs is visually represented by black dots on the corresponding graphs, highlighting their contributions. To assess the robustness of our primary findings, we conducted a noise perturbation analysis by introducing random variations to key input parameters. Specifically, we applied a 10% Gaussian noise to both population data and GBD-derived values across 100 independent iterations.

## 3 Result

### 3.1 Burden of AGN at the global level

The global incidence of AGN decreased by 15.85%, from 627,898 cases (95% UI: 520,122–756,548) in 1990 to 528,368 (95% UI: 446,739–613,211) in 2021 (Table 1). The number of deaths attributed to AGN also decreased from 12,876 (95% UI: 10,020–16,073) in 1990 to 10,761 (95% UI: 7,668–13,463) in 2021 (Table 2). Regarding DALY, the total DALY due to AGN decreased from 567,449 (95% UI: 439,240–687,345) in 1990 to 312,303 (95% UI: 208,981 to 393,514) in 2021. Age-standardized rates of incidence, deaths, and DALY also significantly decreased over the past three decades, suggesting a decrease in the global burden of AGN (Tables 1–3).

**Table 1:** Global and regional trends in acute glomerulonephritis incidence.

**Location**		**ASIR (95% CI)**		**1990–2021 EAPC (95% CI)**	**Incidence number (95% CI)**		**Case change (95% CI)**
		**1990**	**2021**		**1990**	**2021**	
Global	Both	12.28 (10.19, 14.64)	6.62 (5.59, 7.7)	−2.24 (−2.55 to −1.92)	627, 898 (520, 122, 756, 548)	528, 368 (446, 739, 613, 211)	−0.16 (−0.21, −0.11)
Sex	Male	13.10 (10.93, 15.66)	6.93 (5.87, 8.01)	−2.33 (−2.69 to −1.97)	331, 310 (274, 032, 398, 306)	276, 420 (233, 928, 319, 454)	−0.17 (−0.22, −0.12)
	Female	11.52 (9.50, 13.65)	6.33 (5.34, 7.41)	−2.13 (−2.39 to −1.87)	296, 588 (245, 539, 357, 037)	251, 948 (213, 176, 294, 556)	−0.15 (−0.20, −0.10)
SDI	High SDI	5.55 (4.59, 6.63)	4.47 (3.74, 5.28)	−0.61 (−0.74 to −0.48)	48, 699 (40, 307, 57, 940)	51, 821 (43, 582, 60, 792)	0.06 (0.01, 0.13)
	High-middle SDI	21.24 (17.61, 25.66)	7.92 (6.69, 9.26)	−3.67 (−4.13 to −3.22)	228, 574 (187, 628, 278, 030)	110, 743 (94, 075, 129, 518)	−0.52 (−0.55, −0.48)
	Middle SDI	16.51 (13.74, 19.7)	8.17 (6.97, 9.48)	−2.60 (−3.03 to −2.16)	264, 559 (217, 789, 319, 368)	199, 618 (170, 333, 231, 258)	−0.25 (−0.31, −0.18)
	Low-middle SDI	5.57 (4.67, 6.56)	5.94 (5, 6.92)	0.27 (0.20 to 0.34)	62, 886 (51, 593, 75, 525)	113, 198 (95, 102, 133, 275)	0.8 (0.72, 0.89)
	Low SDI	4.98 (4.13, 5.92)	5.03 (4.19, 6.01)	0.01 (−0.02 to 0.03)	22, 784 (18, 419, 27, 796)	52, 542 (42, 294, 64, 492)	1.31 (1.25, 1.36)
Region	Andean Latin America	4.34 (3.39, 5.32)	4.45 (3.51, 5.49)	0.13 (0.05 to 0.20)	1, 713 (1, 283, 2, 150)	2, 912 (2, 299, 3, 582)	0.7 (0.58, 0.84)
	Australasia	2.68 (2.13, 3.27)	2.58 (2.09, 3.12)	−0.14 (−0.18 to −0.11)	536 (432, 648)	803 (655, 972)	0.5 (0.41, 0.6)
	Caribbean	5.21 (4.08, 6.43)	5.42 (4.27, 6.7)	0.12 (0.06 to 0.17)	1, 850 (1, 445, 2, 339)	2, 558 (2, 023, 3, 161)	0.38 (0.3, 0.48)
	Central Asia	18.99 (15.56, 23.19)	17.4 (14.2, 21.38)	−0.33 (−0.41 to −0.24)	12,211 (9,814, 15,144)	17,026 (13,723, 20,925)	0.39 (0.3, 0.52)
	Central Europe	8.85 (7.21, 10.72)	8.28 (6.73, 9.99)	−0.21 (−0.24 to −0.18)	11,003 (9,055, 13,315)	9,011 (7,379, 10,828)	−0.18 (−0.22, −0.13)
	Central Latin America	7.13 (5.68, 8.63)	7.92 (6.51, 9.45)	0.09 (−0.11 to 0.28)	11,123 (8,685, 13,723)	19,876 (16,309, 23,770)	0.79 (0.59, 1.02)
	Central Sub-Saharan Africa	7.68 (6.28, 9.34)	7.76 (6.32, 9.45)	0.07 (0.04 to 0.10)	3,595 (2,850, 4,462)	9,101 (7,224, 11,371)	1.53 (1.43, 1.63)
	East Asia	29.62 (24.36, 35.78)	8.4 (7.15, 9.84)	−4.76 (−5.49 to −4.02)	344,098 (279,311, 421,473)	129,062 (110,595, 149,785)	−0.62 (−0.67, −0.58)
	Eastern Europe	24.82 (21.22, 29)	16.17 (13.71, 18.83)	−1.56 (−1.86 to −1.27)	61,621 (52,722, 72,619)	37,645 (31,829, 44,307)	−0.39 (−0.41, −0.37)
	Eastern Sub-Saharan Africa	7.12 (5.97, 8.37)	6.73 (5.64, 7.98)	−0.25 (−0.29 to −0.22)	11,723 (9,567, 14,242)	25,673 (20,761, 31,490)	1.19 (1.12, 1.25)
	High-income Asia Pacific	9.01 (7.51, 10.78)	10.36 (8.5, 12.45)	0.83 (0.61 to 1.05)	15,514 (13,012, 18,391)	18,456 (15,621, 21,614)	0.19 (0.12, 0.28)
	High-income North America	2.16 (1.73, 2.65)	1.72 (1.52, 1.96)	−0.37 (−0.53 to −0.20)	6,484 (5,195, 7,956)	8,021 (7,004, 9,147)	0.24 (0.09, 0.4)
	North Africa and Middle East	5.73 (4.65, 6.98)	8.1 (6.71, 9.64)	1.26 (1.17 to 1.34)	18,531 (14,795, 22,874)	48,421 (40,019, 58,061)	1.61 (1.42, 1.85)
	Oceania	8.96 (7.08, 11.13)	8.76 (6.94, 10.82)	−0.08 (−0.17 to 0.00)	620 (464, 790)	1,267 (981, 1,573)	1.04 (0.95, 1.15)
	South Asia	3.11 (2.64, 3.64)	3.06 (2.6, 3.59)	−0.05 (−0.11 to 0.00)	32,436 (27,367, 38,423)	57,189 (48,227, 67,757)	0.76 (0.7, 0.83)
	Southeast Asia	13.19 (10.91, 15.75)	9.88 (8.18, 11.76)	−1.09 (−1.18 to −1.01)	63,020 (50,474, 77,514)	66,844 (55,691, 79,444)	0.06 (−0.01, 0.14)
	Southern Latin America	2.27 (1.78, 2.87)	2.58 (2.01, 3.23)	0.37 (0.22 to 0.52)	1,122 (880, 1,421)	1,821 (1,429, 2,290)	0.62 (0.51, 0.8)
	Southern Sub-Saharan Africa	5.84 (4.93, 6.84)	5.6 (4.74, 6.61)	−0.09 (−0.11 to −0.07)	2,905 (2,410, 3,457)	4,499 (3,785, 5,352)	0.55 (0.48, 0.62)
	Tropical Latin America	7.2 (6.11, 8.3)	19.58 (16.57, 23)	3.97 (3.51 to 4.42)	11,307 (9,402, 13,354)	40,968 (35,193, 47,287)	2.62 (2.4, 2.87)
	Western Europe	2.71 (2.17, 3.27)	2.64 (2.12, 3.21)	−0.04 (−0.11 to 0.03)	10,203 (8,337, 12,335)	10,771 (8,759, 12,805)	0.06 (0.01, 0.1)
Western Sub-Saharan Africa	3.43 (2.83, 4.12)	3.5 (2.88, 4.19)	0.04 (−0.03 to 0.11)	6,283 (5,029, 7,690)	16,443 (13,159, 20,189)	1.62 (1.57, 1.65)

**Table 2:** Global and regional trends in acute glomerulonephritis mortality.

**Location**		**ASDR (95% CI)**		**1990–2021 EAPC (95% CI)**	**Death number (95% CI)**		**Case change (95% CI)**
		**1990**	**2021**		**1990**	**2021**	
Global	Both	0.29 (0.23, 0.37)	0.13 (0.09, 0.16)	−2.36 (−2.60 to −2.11)	12,876 (10,020, 16,073)	10,761 (7,668, 13,463)	−0.16 (−0.34, 0.02)
Sex	Male	0.33 (0.24, 0.42)	0.16 (0.10, 0.22)	−2.21 (−2.46 to −1.96)	6,911 (4,743, 9,521)	5,819 (3,459, 8,132)	−0.16 (−0.40, 0.16)
	Female	0.26 (0.19, 0.33)	0.11 (0.09, 0.15)	−2.50 (−2.74 to −2.26)	5,965 (4,536, 7,716)	4,941 (3,942, 6,264)	−0.17 (−0.35, 0.03)
SDI	High SDI	0.03 (0.03, 0.04)	0.05 (0.05, 0.06)	1.95 (0.51 to 3.42)	365 (312, 423)	1,253 (1,045, 1,440)	2.44 (1.93, 2.77)
	High-middle SDI	0.35 (0.28, 0.44)	0.12 (0.09, 0.16)	−3.31 (−3.49 to −3.13)	3,558 (2,873, 4,462)	2,264 (1,717, 2,942)	−0.36 (−0.53, −0.2)
	Middle SDI	0.6 (0.46, 0.77)	0.23 (0.16, 0.29)	−2.75 (−2.99 to −2.52)	7,163 (5,487, 9,006)	5,535 (3,871, 7,053)	−0.23 (−0.42, −0.02)
	Low-middle SDI	0.15 (0.09, 0.21)	0.07 (0.04, 0.09)	−2.41 (−2.46 to −2.36)	1,200 (736, 1,588)	974 (555, 1,329)	−0.19 (−0.36, 0.09)
	Low SDI	0.19 (0.07, 0.44)	0.12 (0.04, 0.23)	−1.51 (−1.61 to −1.41)	586 (225, 1,143)	728 (259, 1,337)	0.24 (−0.15, 0.83)
	Region	Andean Latin America	0.06 (0.04, 0.08)	0.03 (0.02, 0.04)	−2.92 (−4.06 to −1.78)	16 (11, 20)	17 (13, 22)	0.06 (−0.16, 0.41)
		Australasia	0.02 (0.02, 0.03)	0.03 (0.03, 0.04)	2.72 (−0.24 to 5.78)	5 (4, 6)	19 (15, 24)	2.73 (1.86, 3.79)
	Caribbean	0.05 (0.04, 0.06)	0.04 (0.03, 0.05)	−0.39 (−1.39 to 0.62)	15 (12, 18)	20 (16, 25)	0.37 (0.13, 0.64)
	Central Asia	0.33 (0.22, 0.45)	0.1 (0.07, 0.14)	−4.12 (−4.71 to −3.54)	178 (122, 242)	93 (67, 125)	−0.48 (−0.64, −0.2)
	Central Europe	0.07 (0.05, 0.08)	0.11 (0.09, 0.13)	1.11 (−0.94 to 3.19)	89 (73, 107)	227 (189, 270)	1.56 (1.19, 2.06)
	Central Latin America	0.06 (0.06, 0.07)	0.46 (0.39, 0.53)	8.13 (4.55 to 11.83)	56 (49, 64)	1,103 (949, 1,279)	18.7 (16.2, 21.48)
	Central Sub-Saharan Africa	0.22 (0.08, 0.4)	0.14 (0.05, 0.26)	−1.18 (−1.37 to −0.99)	61 (26, 94)	83 (33, 146)	0.36 (−0.08, 1.17)
	East Asia	1 (0.75, 1.28)	0.3 (0.21, 0.4)	−3.34 (−3.50 to −3.19)	8,858 (6,681, 11,386)	5,860 (4,004, 7,736)	−0.34 (−0.54, −0.12)
	Eastern Europe	0.33 (0.29, 0.38)	0.05 (0.04, 0.06)	−7.29 (-8.15 to −6.42)	860 (752, 981)	152 (132, 172)	−0.82 (−0.84, −0.8)
	Eastern Sub-Saharan Africa	0.29 (0.08, 0.7)	0.17 (0.05, 0.38)	−1.64 (−1.74 to −1.54)	295 (97, 613)	349 (115, 708)	0.18 (−0.27, 0.86)
	High-income Asia Pacific	0.04 (0.03, 0.05)	0.03 (0.02, 0.03)	−1.40 (−1.70 to −1.10)	68 (57, 82)	173 (132, 210)	1.53 (1.13, 1.94)
	High-income North America	0.02 (0.02, 0.03)	0.09 (0.07, 0.1)	5.90 (2.86 to 9.04)	91 (78, 104)	617 (515, 719)	5.77 (5.15, 6.39)
	North Africa and Middle East	0.12 (0.07, 0.21)	0.07 (0.05, 0.09)	−1.00 (−1.34 to −0.65)	228 (152, 370)	301 (194, 411)	0.32 (−0.13, 1.01)
	Oceania	0 (0, 0.01)	0 (0, 0)	−2.12 (−2.16 to −2.09)	0 (0, 0)	0 (0, 0)	0.15 (−0.35, 1.32)
	South Asia	0.05 (0.02, 0.08)	0.02 (0.01, 0.03)	−3.02 (−3.18 to −2.86)	296 (181, 510)	254 (148, 429)	−0.14 (−0.34, 0.18)
Southeast Asia	0.29 (0.16, 0.46)	0.12 (0.06, 0.17)	−2.76 (−2.88 to −2.64)	1,201 (644, 1,752)	724 (388, 1,048)	−0.4 (−0.56, −0.01)
Southern Latin America	0.01 (0.01, 0.01)	0.01 (0.01, 0.01)	1.33 (−0.67 to 3.37)	4 (3, 5)	10 (8, 11)	1.4 (0.89, 2.09)
Southern Sub-Saharan Africa	0.07 (0.05, 0.11)	0.07 (0.05, 0.1)	0.44 (0.19 to 0.68)	30 (22, 45)	47 (33, 65)	0.55 (0.14, 1.09)
Tropical Latin America	0.15 (0.12, 0.17)	0.04 (0.03, 0.04)	−4.48 (−5.32 to −3.64)	183 (155, 210)	84 (73, 95)	−0.54 (−0.61, −0.48)
Western Europe	0.01 (0.01, 0.02)	0.02 (0.02, 0.03)	2.08 (−0.30 to 4.52)	81 (68, 94)	265 (217, 310)	2.28 (1.96, 2.61)
Western Sub-Saharan Africa	0.22 (0.06, 0.56)	0.14 (0.04, 0.3)	−1.08 (−1.26 to −0.90)	261 (83, 560)	363 (119, 679)	0.39 (−0.06, 1.14)

**Table 3:** Global and regional trends in acute glomerulonephritis DALY.

**Location**		**Age-standardized DALY rate (95% CI)**		**1990−2021 EAPC (95% CI)**	**DALY number (95% CI)**		**Case change (95% CI)**
		**1990**	**2021**		**1990**	**2021**	
Global	Both	11.23 (8.73, 13.72)	3.83 (2.55, 4.83)	−3.26 (−3.43 to −3.09)	567,449 (439,240, 687,345)	312,303 (208,981, 393,514)	−0.45 (−0.58, −0.32)
Sex	Male	12.50 (8.39, 16.77)	4.42 (2.41, 6.12)	−3.15 (−3.33 to −2.96)	311,630 (205,700, 409,470)	174,084 (94,539, 241,993)	−0.44 (−0.60, −0.22)
	Female	10.05 (7.90, 12.97)	3.31 (2.66, 4.42)	−3.37 (−3.53 to −3.22)	255,819 (203,394, 332,617)	138,219 (111,898, 182,590)	−0.46 (−0.57, −0.33)
SDI	High SDI	0.91 (0.78, 1.09)	1.13 (0.99, 1.28)	1.04 (−0.30 to 2.39)	9,110 (7,813, 10,857)	21,340 (18,454, 24,181)	1.34 (0.95, 1.57)
	High-middle SDI	13.38 (10.82, 16.89)	3.49 (2.65, 4.54)	−4.31 (−4.47 to −4.14)	140,424 (113,781, 177,216)	58,766 (44,386, 76,341)	−0.58 (−0.69, −0.47)
	Middle SDI	21.19 (16.25, 26.29)	6.27 (4.24, 7.96)	−3.57 (−3.76 to −3.38)	321,162 (247,394, 394,368)	158,469 (107,075, 202,048)	−0.51 (−0.62, −0.37)
	Low-middle SDI	5.69 (3.49, 7.43)	2.21 (1.24, 2.97)	−2.97 (−3.03 to −2.91)	65,684 (39,873, 83,677)	38,316 (21,051, 51,700)	−0.42 (−0.59, −0.14)
	Low SDI	6.7 (2.54, 13.52)	3.76 (1.35, 6.86)	−1.76 (−1.86 to −1.66)	30,866 (11,898, 50,955)	35,236 (13,250, 59,201)	0.14 (−0.26, 0.79)
Region	Andean Latin America	2.22 (1.49, 2.73)	0.86 (0.65, 1.13)	−3.67 (−4.73 to −2.59)	873 (601, 1,068)	530 (398, 698)	−0.39 (−0.54, −0.16)
	Australasia	0.53 (0.44, 0.62)	0.64 (0.51, 0.79)	2.01 (−0.77 to 4.86)	117 (98, 138)	316 (250, 386)	1.71 (1.18, 2.34)
	Caribbean	1.98 (1.56, 2.35)	1.38 (1.07, 1.74)	−0.62 (−1.46 to 0.22)	676 (534, 804)	666 (515, 830)	−0.02 (−0.21, 0.23)
	Central Asia	12.98 (9.19, 17.24)	4.08 (2.93, 5.42)	−4.18 (−4.78 to −3.59)	8,088 (5,830, 10,517)	3,938 (2,827, 5,219)	−0.51 (−0.67, −0.28)
	Central Europe	2.17 (1.78, 2.64)	2.99 (2.54, 3.52)	0.37 (−1.55 to 2.33)	2,855 (2,358, 3,463)	5,165 (4,362, 6,125)	0.81 (0.54, 1.15)
	Central Latin America	1.7 (1.49, 1.92)	11.27 (9.77, 13.05)	7.76 (4.29 to 11.34)	2,171 (1,907, 2,454)	28,160 (24,399, 32,530)	11.97 (10.32, 13.82)
	Central Sub-Saharan Africa	6.82 (2.89, 11.09)	3.86 (1.56, 6.81)	−1.57 (−1.76 to −1.37)	3,083 (1,455, 4,314)	3,606 (1,469, 5,805)	0.17 (−0.24, 0.97)
	East Asia	33.56 (25.36, 42.85)	8.4 (5.64, 11.23)	−4.08 (−4.20 to −3.96)	365,301 (276,089, 463,654)	153,611 (104,422, 204,226)	−0.58 (−0.7, −0.44)
	Eastern Europe	13.77 (12.1, 15.66)	1.92 (1.69, 2.16)	−7.54 (-8.33 to −6.74)	33,950 (29,750, 38,655)	5,167 (4,532, 5,812)	−0.85 (−0.87, −0.83)
	Eastern Sub-Saharan Africa	9.56 (3.02, 20.79)	5.08 (1.67, 10.35)	−1.99 (−2.08 to −1.91)	15,327 (5,399, 27,555)	16,165 (5,731, 30,105)	0.05 (−0.38, 0.83)
	High-income Asia Pacific	0.72 (0.61, 0.86)	0.5 (0.42, 0.6)	−1.53 (−1.82 to −1.24)	1,343 (1,140, 1,596)	2,394 (1,910, 2,878)	0.78 (0.5, 1.06)
	High-income North America	0.48 (0.42, 0.54)	1.6 (1.38, 1.84)	5.75 (2.78 to 8.81)	1,659 (1,444, 1,885)	9,977 (8,549, 11,555)	5.01 (4.49, 5.52)
	North Africa and Middle East	3.63 (2.46, 5.74)	1.99 (1.26, 2.69)	−1.19 (−1.57 to −0.81)	10,502 (7,578, 14,704)	10,420 (6,511, 14,102)	−0.01 (−0.32, 0.56)
	Oceania	0.11 (0.04, 0.26)	0.06 (0.04, 0.12)	−1.68 (−1.71 to −1.64)	6 (3, 12)	8 (5, 14)	0.42 (−0.1, 1.09)
	South Asia	1.46 (0.91, 2.48)	0.53 (0.32, 0.86)	−3.42 (−3.58 to −3.25)	13,398 (8,796, 21,021)	8,650 (5,341, 14,048)	−0.35 (−0.52, −0.05)
	Southeast Asia	15.48 (7.98, 22.12)	5.59 (3.03, 7.99)	−3.08 (−3.19 to −2.97)	79,675 (39,372, 115,392)	36,163 (19,486, 52,079)	−0.55 (−0.69, −0.2)
	Southern Latin America	0.28 (0.21, 0.36)	0.31 (0.26, 0.38)	0.87 (−0.95 to 2.72)	135 (100, 175)	234 (194, 278)	0.74 (0.39, 1.21)
	Southern Sub-Saharan Africa	3.3 (2.39, 4.92)	2.91 (2.06, 4.09)	0.15 (−0.09 to 0.38)	1,724 (1,248, 2,471)	2,249 (1,579, 3,172)	0.3 (−0.06, 0.81)
	Tropical Latin America	7.19 (6.1, 8.34)	1.34 (1.15, 1.55)	−5.26 (−5.91 to −4.60)	11,359 (9,581, 13,249)	2,966 (2,576, 3,394)	−0.74 (−0.78, −0.7)
	Western Europe	0.31 (0.27, 0.35)	0.42 (0.37, 0.48)	1.39 (−0.79 to 3.63)	1,573 (1,352, 1,809)	3,818 (3,239, 4,397)	1.43 (1.22, 1.63)
Western Sub-Saharan Africa	7.5 (2.37, 16.62)	4.43 (1.44, 8.42)	−1.39 (−1.55 to −1.22)	13,633 (4,603, 24,491)	18,100 (6,383, 30,103)	0.33 (−0.14, 1.17)

### 3.2 Burden of AGN at the regional level

In accordance to the SDI categorization, in 2021 middle SDI region exhibited the highest ASIR (8.17, 95% UI: 6.97–9.48), ASDR (0.23; 95% UI: 0.16–0.29) and age-standardized DALY rate (6.27, 95% UI: 4.24–7.96) (Tables 1–3). Conversely, the high SDI region showed the lowest ASIR (4.47, 95% UI: 3.74–5.28), ASDR (0.05, 95% UI: 0.05–0.06), and age-standardized DALY rate (1.13, 95% UI: 0.99–1.28) (Tables 1–3). Indicating disparity of distribution and clinical outcome of AGN in different SDI regions. From 1990 to 2021, most SDI regions showed a decreased burden of AGN. Specifically, ASIR in high, high-middle, and middle SDI decreased compared to 1990. The largest decrease of ASIR was found in the high-middle SDI region, with an EAPC of −3.67 (95% CI: −4.13 to −3.22). The low-middle SDI region exhibited the highest increase in ASIR, with an EAPC of 0.27 (95% CI: 0.20–0.34) (Table 1). ASDR and age-standardized DALY rate of AGN were decreased in all of the SDI regions except for the high SDI region. The largest decrease of ASDR was also found in the high-middle SDI region, with an EAPC of −3.31 (95% CI: −3.49 to 3.13). Moreover, the high-middle SDI region had the largest decrease in age-standardized DALY rate with an EAPC of −4.31 (95% CI: −4.47 to 4.41). The high SDI region was the only SDI region that showed increased ASDR and age-standardized DALY rate, with an EAPC of 1.95 (95% CI: 0.51–3.42) and 1.04 (95% CI: −0.30 to 2.39), respectively (Tables 2, 3).

Among 21 GBD regions, Tropical Latin America (19.58 per 100,000; 95% UI: 16.57–23) had the highest ASIR of AGN, followed by Central Asia and Eastern Europe (Table 1). Conversely, regions with the lowest ASIR were High-income North America (1.72 per 100,000; 95% UI: 1.52–1.96) (Table 1). For ASDR of AGN, the highest ASDR in 2021 was found in Central Latin America (0.46 per 100,000; 95% UI: 0.39–0.53), followed by East Asia and Eastern Sub-Saharan Africa (Table 2). On the other hand, Oceania (0 per 100,000; 95% UI: 0–0), Southern Latin America (0.01 per 100,000; 95% UI: 0.01–0.01) and South Asia (0.02 per 100,000; 95% UI: 0.01–0.03) exhibited lowest ASDR in 2021 (Table 2). Moreover, Central Latin America (11.27 per 100,000; 95%UI: 9.77–13.05) had the highest age-standardized DALY rate, while Oceania (0.06 per 100,000; 95% UI: 0.04–0.12) had the lowest age-standardized DALY rate. From 1990 to 2021, the ASIR of AGN increased in nine regions, accounting for nearly half of the GBD regions. The highest increase of ASIR was found in Tropical Latin America (EAPC = 3.97; 95% UI: 3.51–4.42), followed by North Africa and Middle East (EAPC = 1.26; 95% UI: 1.17–1.34) and High-income Asia Pacific (EAPC = 0.83; 95% UI: 0.61–1.05). Seven saw an increase in ASDR, with Central Latin America exhibiting the highest increase in ASDR (EAPC = 8.13; 95% UI: 4.55–11.83), followed by High-income North America (EAPC = 5.90; 95% UI: 2.86–9.04) and Australasia (EAPC = 2.72; 95% UI: −0.24 to 5.78). Largest decrease of ASDR occurred in Eastern Europe (EAPC = −7.29; 95% UI: −8.15 to −6.42), Tropical Latin America (EAPC = −4.48; −5.32 to −3.64), and Central Asia (EAPC = −4.12; 95% UI: −4.17 to −3.54). The changing trend in age-standardized DALY rates was similar to ASDR. The highest increase of age-standardized DALY rates was also seen in Central Latin America (EAPC = 7.76; 95% UI: 4.29–11.34), High-income North America (EAPC = 5.75; 95% UI: 2.78–8.81), and Australasia (EAPC = −0.77 to 4.86). Correspondingly, the largest decrease in age-standardized DALY rates was also observed in Eastern Europe, Tropical Latin America, and Central Asia (EAPC = −4.18; 95% UI: −4.78 to −3.59).

### 3.3 Burden of AGN at the national (regional) level

**Figure 1:**
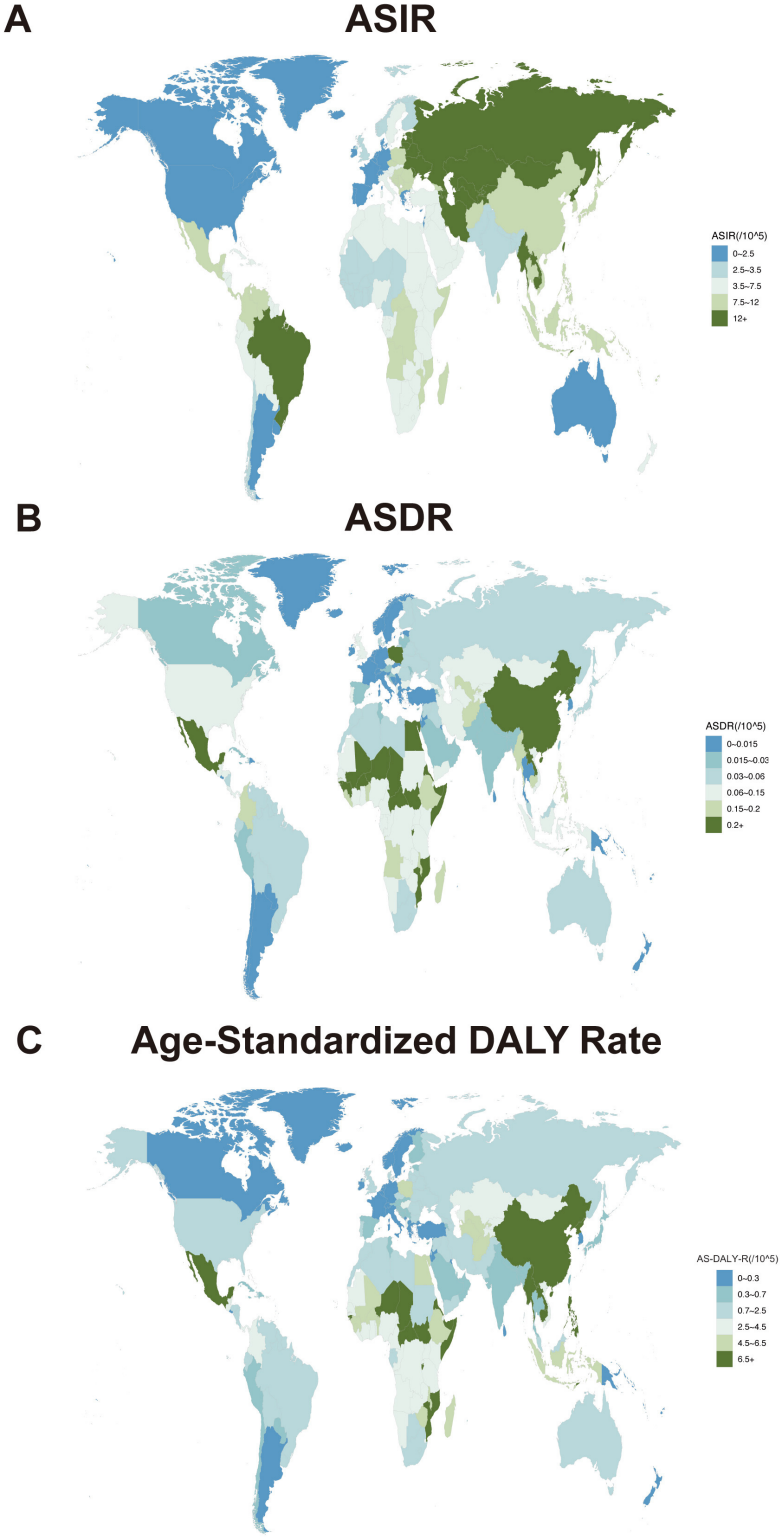
Age-standardized rates of acute glomerulonephritis per 100,000 population in different countries and territories. **(A)** ASIR of acute glomerulonephritis in 2021. **(B)** ASDR of acute glomerulonephritis in 2021. **(C)** Age-standardized DALY rate of acute glomerulonephritis in 2021.

The burden of AGN at the national (regional) level was also analyzed (Figure 1). In 2021, the highest ASIRs of AGN were observed in the Democratic People's Republic of Korea (31.89; 95% UI: 27.03–37.75), followed by Taiwan (Province of China) and Uzbekistan (Figure 1A and Supplementary Table S1). Conversely, the lowest ASIR of AGN was observed in Greece (0.83; 95% UI: 0.61–1.04) (Figure 1A and Supplementary Table S2). Mexico (0.80; 95% UI: 0.68–0.93) exhibited the highest ASDR, while Norway, Barbados, and Guam (ASDR <0.01) had the lowest ASDR in 2021 (Figure 1B and Supplementary Tables S3, S4). Mexico (19.31; 95% UI: 16.66–22.42) also had the highest age-standardized DALY rate, followed by the Democratic People's Republic of Korea and the Lao People's Democratic Republic (Figure 1C and Supplementary Table S5). Last but not least, Norway (0.012; 95% UI: 0.007–0.018), Barbados (0.019; 95% UI: 0.012–0.03) and Greenland (0.023; 95% UI: 0.016–0.034) exhibited the lowest age-standardized DALY rate in 2021 (Figure 1C and Supplementary Table S6).

### 3.4 Age and sex patterns of AGN

**Figure 2:**
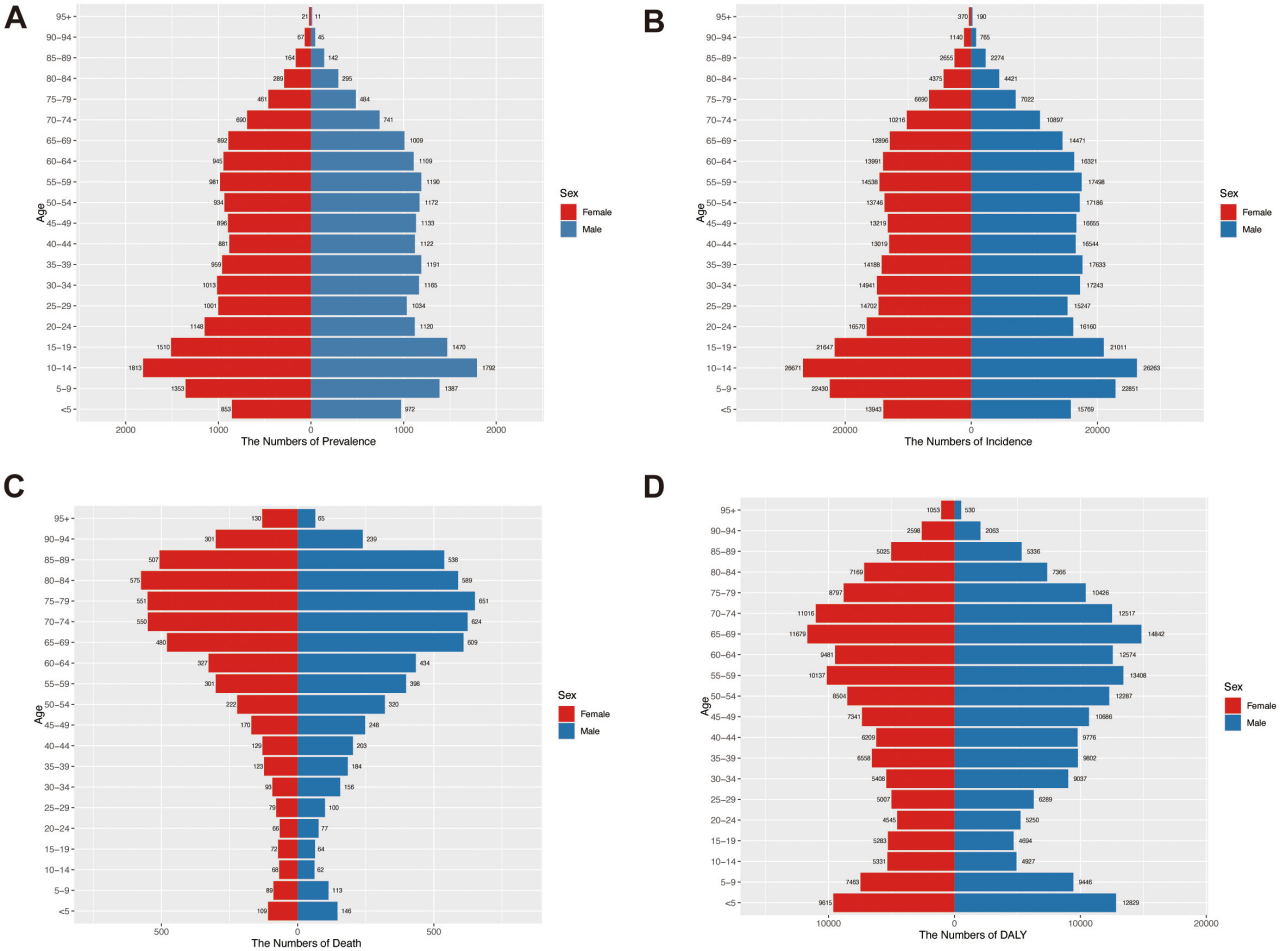
Age-specific numbers of prevalence, incidence, death, and DALY of acute glomerulonephritis by sex in 2021. **(A)** Number of prevalence in different age groups. **(B)** Number of incidents by age groups. **(C)** Number of deaths by age groups. **(D)** Number of DALY in different age groups.

In 2021, the prevalence and incidence of AGN showed a similar age distribution pattern. The Number of both prevalence and incidence peaked at the 10–14 age group, and the rate of both prevalence and incidence showed a bimodal distribution pattern, peaking at age groups 10–14 and 85–89, with males significantly higher than females (Figures 2A, B and Supplementary Figures S1A, B). This bimodal age distribution is consistent with the divergent disease mechanisms: streptococcal-driven pathology in children vs. autoimmune/metabolic etiologies in the older adult. Thus, pediatric AGN demands targeted infection control, and older adults with unexplained AKI should maintain a high index of suspicion for AGN. The number of deaths due to AGN peaked in the age group 75–79. The rate of death increases as age grows, and males have a higher rate of death due to AGN (Figure 2C and Supplementary Figure S1C). Regarding DALY, in 2021, the number of DALY peaked in <5 and 65–69 age group, with males having higher than females. Similar to the death rate, the rate of DALY also increased as age grows, and the DALY rate of males was significantly higher than that of females (Figure 2D and Supplementary Figure S1D).

**Figure 3:**
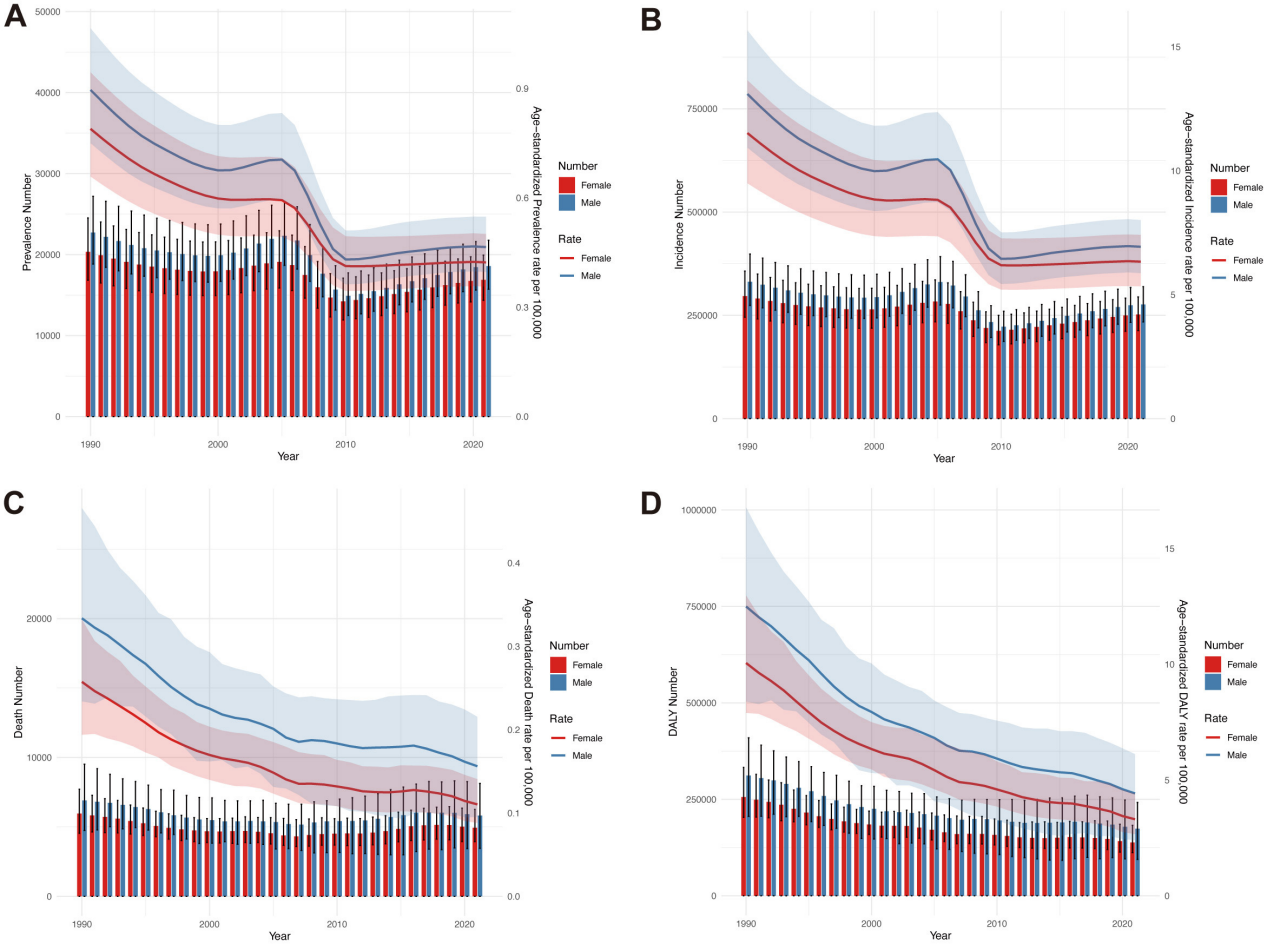
Comparison of full-age cases and age-standardized rates of global prevalence, incidence, death, and DALY of acute glomerulonephritis from 1990 to 2021. **(A)** Number of prevalence and ASPR. **(B)** Number of incidents and ASIR. **(C)** Number of deaths and ASDR. **(D)** Number of DALYs and age-standardized DALY rate.

A trend change of the global burden of AGN from 1990 to 2021 was conducted. The global number and ASR of prevalence and incidence decreased from 1990 to 2000, with males having higher rates than females. This may be caused by androgen-driven immune hyperreactivity and occupational exposures (e.g., streptococcal infections in agrarian work) that disproportionately affect males. Between 2000 and 2010, the prevalence and incidence of AGN experienced a temporary increase from 2000 to 2005, reaching a peak in 2005. A possible explanation is the occurrence of localized or global outbreaks of group A streptococcal infections, which are a major trigger for post-infectious AGN (26). Moreover, improved diagnostic capabilities, particularly in middle- and high-income countries, and publication of clinical practice guidelines in the early 2000s might contribute to the elevation at this period of time (27, 28). Prevalence and incidence remained relatively stable after 2010 (Figures 3A, B). The number of deaths due to AGN remained relatively stable from 1990 to 2021. However, from 1990 to 2021, ASDR due to AGN showed a declining trend, with males consistently higher than females (Figure 3C). Similarly, the age-standardized DALY rate also declined from 1990 to 2021. Age-standardized DALY rate of males was also higher than that of females (Figure 3D).

### 3.5 Association of AGN with sociodemographic index

**Figure 4:**
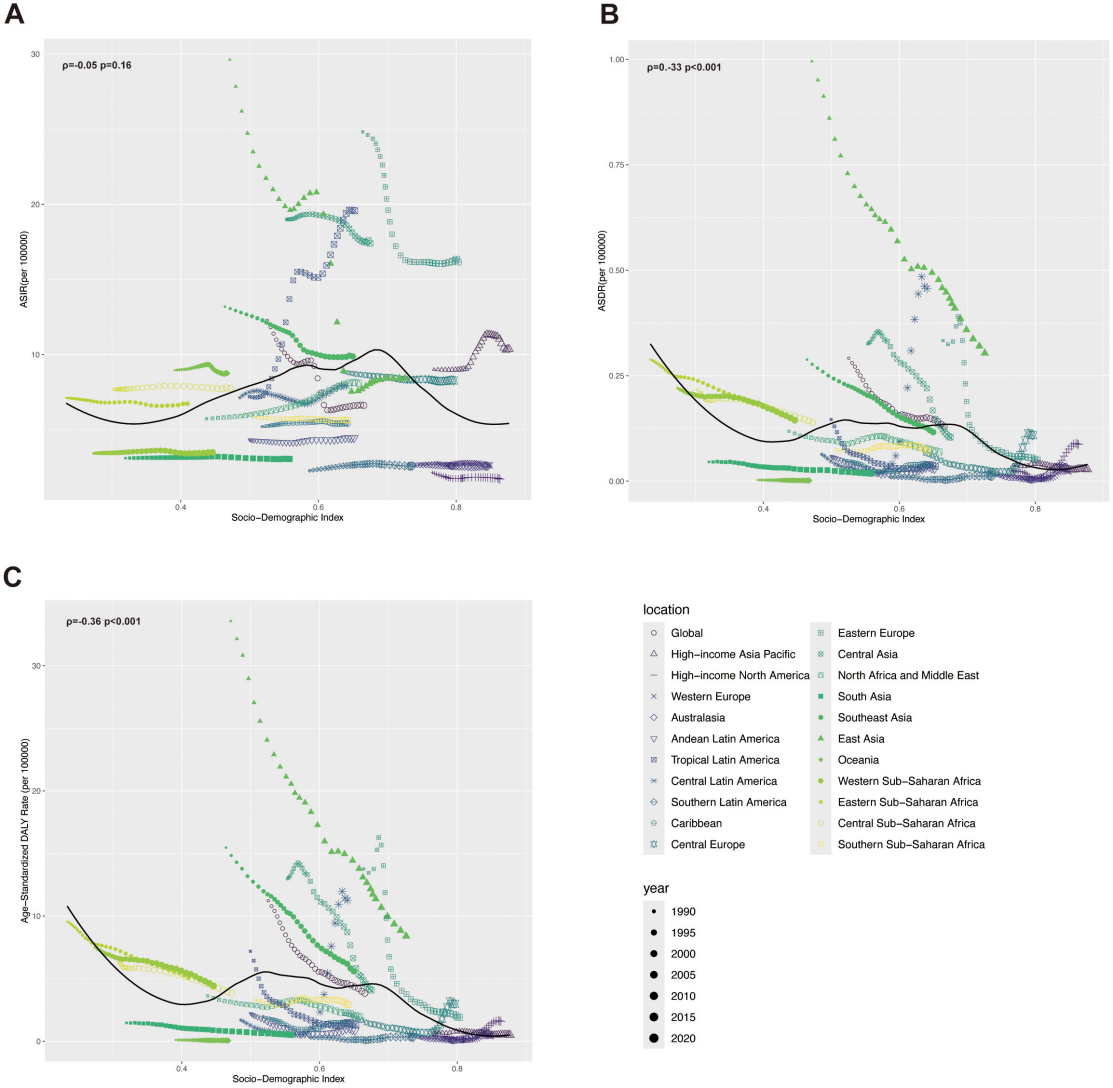
ASRs of incidence, death, and DALY of acute glomerulonephritis and SDI from 1990 to 2021, by GBD regions. **(A)** ASIR in GDB regions by SDI. **(B)** ASDR in GDB regions by SDI. **(C)** Age-standardized DALY rate in GDB regions by SDI.

The Correlation between ASIR, age-standardized DALY rate, and SDI was investigated at the regional and national levels (Figure 4 and Supplementary Figure S2). Regionally, the ASIR showed an inverse V-shaped relationship with SDI. When SDI exceeded 0.7, the ASIR of AGN was negatively associated with SDI, underscoring the transition from infection-dominated AGN in low-SDI settings to metabolic/autoimmune etiologies in high-SDI regions. Regionally, the ASIR of AGN was higher than expected in areas such as East Asia, Tropical Latin America, and Central Asia. Conversely, regions like Southern Latin America, Australasia, and high-income North America had ASIRs lower than expected (Figure 4A). The correlation between SDI and ASIR at the national level of different countries was similar to that at the regional level, with ASIR of countries such as North Korea, Uzbekistan, and Brazil higher than expected (Supplementary Figure S2A). For ASDR at the regional level, ASDR was negatively correlated with SDI when SDI was <0.4 and >0.7, and was unaffected by SDI between 0.4 and 0.7. Areas such as Central Latin America, East Asia, and Central Europe had ASDR lower than expected. Conversely, Oceania, South Asia, and Southern Latin America had ASDR lower than expected (Figure 4B). Nationally, the ASDR of AGN exhibited a negative correlation with SDI, and countries like Mexico, North Korea, and China had ASDR values significantly higher than expected (Supplementary Figure S2B). The correlation of age-standardized DALY rate of AGN and SDI was similar to ASDR (Figure 4C and Supplementary Figure S2C). Regionally, Eastern Europe, Central Latin America, and East Asia had a higher burden than expected, whereas regions such as Oceania, South Asia, and Southern Latin America exhibited a burden less than expected (Figure 4C). At the national level, North Korea, Laos, and Timor-Leste had age-standardized DALY rates that were significantly higher than expected (Supplementary Figure S2C).

### 3.6 Decomposition analysis

**Figure 5:**
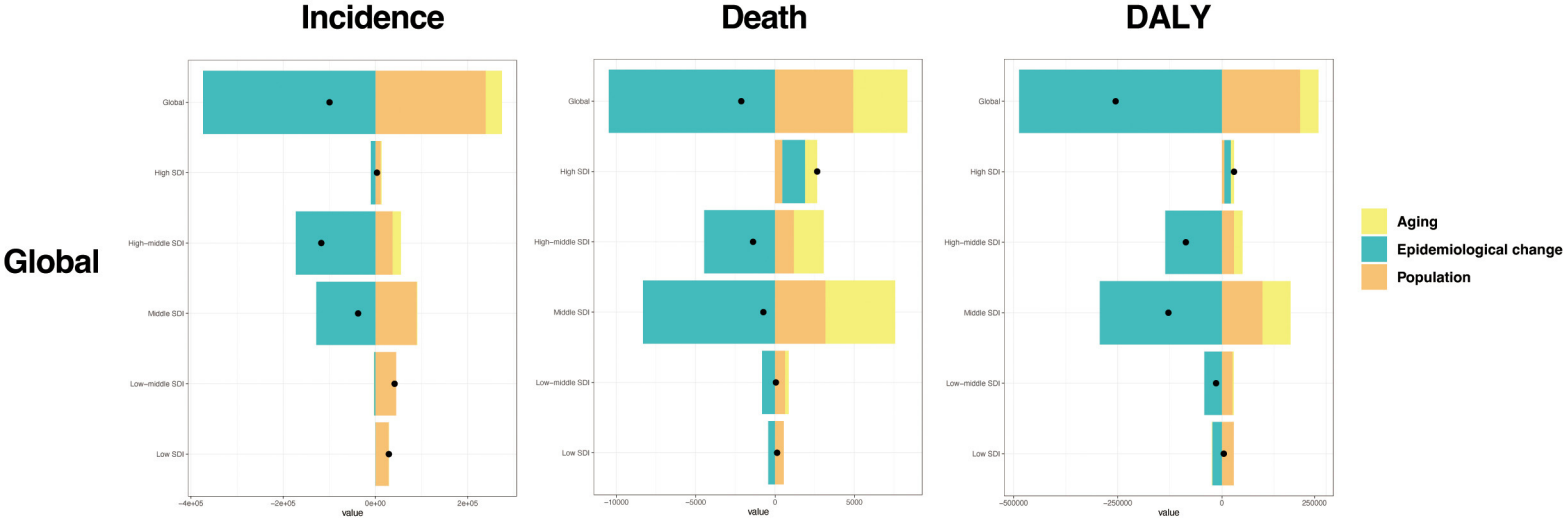
Decomposition analysis of global acute glomerulonephritis ASRs from 1990 to 2021. Black dots in each column indicate the overall difference of each estimate from 1990 to 2021. A positive overall difference means the estimate has increased from 2021 to 1990, while a negative overall difference means the estimate has declined from 2021 to 1990.

Decomposition analysis was conducted to investigate the proportion of contributions of aging, epidemiological changes, and population growth to the burden of AGN from 1990 to 2021 (Figure 5). Epidemiological change (−375.3%) contributed negatively to the increase of global incidence, while aging (+35.36%) and population growth (+239.94%) contributed positively. For the death number, epidemiological change also contributed negatively (−494.69%), while aging (+161.51%) and population growth (+233.17%) contributed positively to the increase of deaths due to AGN. The influence of different factors on the change in global DALY was similar to deaths, with epidemiological changes contributing to a 190.78% reduction in DALY. The contribution of different factors to incidence, death, and DALY in different SDI regions is largely similar to global, but with slight differences. Specifically, aging growth negatively impacts the incidence of AGN in the low-middle SDI region (−1.97%). Epidemiological changes positively contribute to the growth of deaths (+53.75%) and DALY (51.95%) due to AGN in the high SDI region. The proportion of contributions from aging, epidemiological changes, and population growth to incidence, death, and DALY in males and females was similar to the global pattern, except for incidence in the low-middle SDI region. In this region, epidemiological changes contribute positively in males while contributing negatively in females (Figure 5 and Supplementary Figure S3). A noise perturbation analysis was conducted to assess the robustness of our primary findings. The results demonstrated that the directionality of effect estimates remained consistent with the original analysis in all iterations. Although the magnitude of effect sizes showed minor fluctuations, no substantive changes in statistical significance or qualitative conclusions were observed. This suggests that our model is resilient to potential measurement errors or variability within the observed range of input data. A detailed distribution of perturbed outcomes is included in Supplementary Table S7.

### 3.7 Prediction of global disease burden of AGN

**Figure 6:**
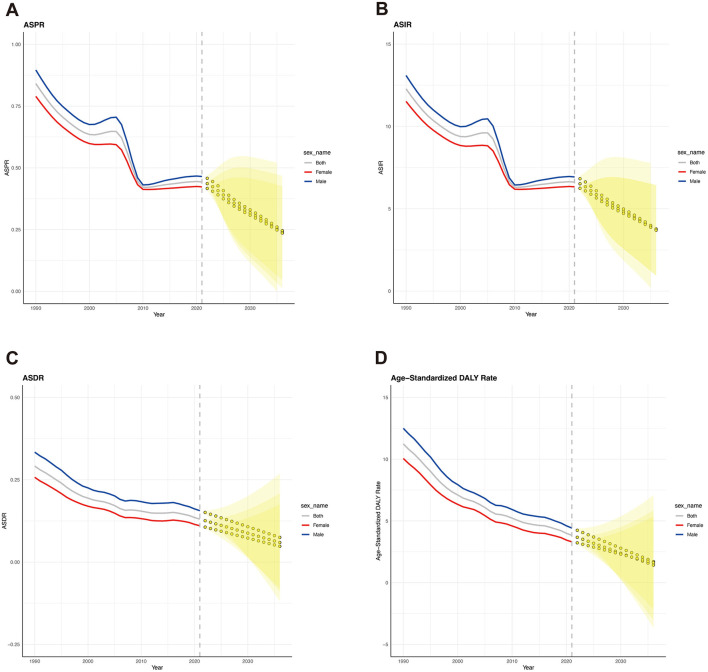
Forecasted trends of global ASRs for acute glomerulonephritis over the next 15 years (2022-2036). **(A)** Forecasted trend of ASPR. **(B)** Forecasted trend of ASIR. **(C)** Forecasted trend of ASDR. **(D)** Forecasted trend of age-standardized DALY rate. Gray line: the true trend of the global age-standardized rates. Blue line: the true trend of the age-standardized rates of males. Red line: the true trend of the age-standardized rates of females. Yellow dotted lines and shaded regions: the predicted trends and their 95% CI.

We used an ARIMA machine learning model to predict the disease burden of AGN in the future. The overall disease burden of AGN is projected to continue decreasing from 2021 to 2036. Although the predicted burden of males is still higher than that of females, the disparity between the two will become smaller (Figures 6A–D). The global ASIR of AGN in 2036 is predicted to be 3.72 per 100,000 people, reflecting a 43.81% decrease (Figure 6B). Both males and females are expected to decrease in ASIR by 2036, with males predicted to reach 3.78 per 100,000 people (a 45.45% decrease) and females predicted to reach 3.69 (a 41.71% decrease). For ASDR due to AGN, it is also predicted to decline to 0.06 (53.84%) by 2036. Last but not least, the age-standardized DALY rate is expected to decrease to 1.42 in 2036 (62.92%). Specifically, the age-standardized DALY rates for males are predicted to be 1.71 (61.31% decline) and 1.62 for females (49.69% decline) (Figure 6D).

## 4 Discussion

This study provides a comprehensive analysis of the global, regional, and national burden of AGN from 1990 to 2021 and projects its burden through 2036. Key findings reveal a substantial global decline in ASIR, ASDR, and age-standardized DALY rates over the past three decades. However, these improvements are not uniformly distributed, with certain regions and populations continuing to face significant challenges.

Based on data from GBD 2019, Guo et al. reported a global decline in AGN burden through 2019; our study extends this analysis to 2021 and projects trends to 2036 (4). Notably, our findings confirm the continued decline in age-standardized incidence rates and DALY rates through 2021, suggesting sustained progress even during the COVID-19 pandemic. Furthermore, our study incorporates decomposition analysis to disentangle the contributions of population growth, aging, and epidemiological changes. For example, we demonstrate that while epidemiological improvements drove global declines, aging populations offset progress in low-middle SDI regions. This granular approach provides novel insights into the drivers of AGN burden. Sensitivity analysis confirmed the robustness of our findings, which ensures the reliability of the contributing factors. This reliability allows policymakers and researchers to confidently use these findings to inform targeted public health interventions and strategies aimed at reducing the disease burden. Notably, between 2000 and 2005, the ASIR and ASDR of AGN experienced a temporary elevation before declining thereafter. This temporary rise could be attributed to several factors, including improvements in diagnostic capabilities during the early 2000s, particularly in middle- and high-income countries (29). The publication of clinical practice guidelines, along with enhanced access to healthcare services, including laboratory diagnostics for renal conditions, likely contributed to improved detection and reporting of AGN cases during that period (27, 28). Another possible explanation is the occurrence of localized or global outbreaks of infectious diseases, particularly group A streptococcal infections, which are a major trigger for post-infectious AGN (26). Such outbreaks may have temporarily increased the incidence and severity of AGN cases during this period (30). Mortality trends showed a rather consistent decrease. The DALY burden mirrored the trend, with the age-standardized DALY rate dropping by 65.87% over three decades. Despite these encouraging trends, regions with middle and low-middle SDI values continued to exhibit the highest burden in 2021, with Tropical Latin America, Central Asia, and Eastern Europe leading in ASIR. The decomposition analysis highlights the contributions of demographic and epidemiological shifts to the observed changes. Aging and population growth contributed positively to AGN burden, while epidemiological improvements drove reductions in incidence, mortality, and DALY rates. Interestingly, the high-SDI region was an outlier, showing an increase in ASDR and age-standardized DALY rate. This may be caused by the improved diagnostics in high-SDI areas, AGN, and inflated mortality estimates. However, the persistence of high burdens in middle- and low-middle-SDI regions highlights the ongoing need for interventions tailored to local contexts. Efforts should prioritize strengthening healthcare systems to improve early diagnosis and management of AGN (31), particularly in regions with limited resources. Addressing the socioeconomic determinants of health, such as poverty, inadequate sanitation, and limited access to healthcare, is critical to reducing regional disparities. Moreover, targeted education campaigns could raise awareness about AGN risk factors and symptoms, encouraging timely healthcare-seeking behavior.

The findings of this study align with previous research that highlights the impact of improved healthcare, sanitation, and infection control in reducing the global burden of AGN (4). Earlier studies have also noted the disproportionate burden of infectious diseases, including AGN, in low- and middle-income countries, consistent with the trends observed in our analysis (4). However, this inconsistency persists in the 2021 data across different regions. The regional variations, particularly the unexpectedly high burden in middle-SDI regions, reinforce the need for targeted interventions in these areas. Environmental factors, such as poor sanitation, overcrowding, and limited access to clean water, likely exacerbate streptococcal infections in regions like Tropical Latin America and Central Asia, driving higher ASIR (32). Genetic predispositions, including specific HLA alleles, may also increase susceptibility to AGN in certain populations, such as those in East Asia, contributing to regional disparities (5). These environmental and genetic factors, not fully accounted for in this study, highlight the complexity of AGN epidemiology and the need for region-specific strategies. The significant reductions in AGN burden over time underscore the effectiveness of global health initiatives, such as improved access to antibiotics, vaccination programs, and enhanced diagnostic capabilities (33–35). However, the persistence of high burdens in middle- and low-middle-SDI regions highlights the ongoing need for interventions tailored to local contexts. While baseline projections indicate a steady decline in AGN burden, combining vaccination, healthcare access, and precision therapies could significantly enhance the progress. Prioritizing these interventions, particularly in high-burden regions, would maximize lives saved and advance health equity. Addressing the AGN burden also has broader implications for achieving the Sustainable Development Goals (SDGs), particularly SDG 3 (Good Health and WellBeing), which aims to reduce non-communicable diseases and achieve universal health coverage (UHC). By ensuring equitable access to diagnostics, treatment, and preventive care, UHC can mitigate AGN disparities.

This study has several limitations. First, the reliance on GBD estimates may introduce biases due to variations in data quality across regions. Specifically, regions with robust healthcare infrastructure and effective reporting systems likely provide more accurate data, whereas those with limited resources may underreport cases, potentially underestimating the burden of acute glomerulonephritis. Moreover, GBD employs modeling to address data gaps, which introduces additional uncertainty. These models rely on assumptions that may not fully capture regional differences, which can lead to biased estimates. Second, the ARIMA model used for future projections assumes that historical trends observed from 1990 to 2021 will persist through 2036. However, this assumption may not hold, as unanticipated changes, such as advancements in healthcare, shifts in public health policies, or external events (e.g., pandemics) could disrupt these trends, limiting the accuracy of the forecasts (36). Consequently, the predicted estimates should be interpreted with caution. Furthermore, this study does not account for all potential contributors to AGN burden, such as environmental and genetic factors, which may influence regional and national differences. Future analyses could integrate these variables to provide a more nuanced understanding of the relationship.

To build on the findings of this study, further research is needed to investigate the drivers of AGN burden in regions exhibiting unexpected trends, such as the high-SDI region. Longitudinal and case-control studies could provide insights into the role of specific risk factors, including autoimmune and systemic diseases. Furthermore, there is a need for more granular analyses to identify within-country disparities and target high-risk populations. Future projections should incorporate scenario-based modeling to account for the impact of public health interventions and policy changes.

## 5 Conclusion

This study demonstrated significant reductions in the global burden of AGN from 1990 to 2021, highlighting the positive impact of advancements in healthcare, sanitation, and public health initiatives. However, substantial disparities persist, with low- and middle-income countries facing disproportionately high burdens due to limited healthcare resources and a high prevalence of infectious diseases. Unique trends in high-SDI regions, such as increasing mortality and DALY rates, need further investigation into potential contributions from autoimmune and systemic diseases. Healthcare systems should prioritize training for early AGN detection, integrate care into streptococcal control programs, and fund hygiene campaigns to reduce infections cost-effectively. These efforts align with the SDGs and UHC, ensuring equitable care. Future research should include cohort studies on autoimmune contributions in high-SDI regions, genomic studies to identify regional risk factors, and trials to evaluate interventions. Subnational analyses can address within-country disparities. Targeted policies and research will advance health equity.

## Data Availability

The original contributions presented in the study are included in the article/Supplementary material, further inquiries can be directed to the corresponding authors.

## Supplementary material

Supplementary Figure S1 Age-specific rate of prevalence, incidence, death, and DALY of acute glomerulonephritis by sex in 2021. **(A)** Rate of prevalence in different age groups. **(B)** Rate of incidence in different age groups. **(C)** The rate of death in different age groups. **(D)** Rate of DALY in different age groups.

Supplementary Figure S2 ASRs of incidence, death, and DALY of acute glomerulonephritis and SDI from 1990 to 2021, by country and territory. **(A)** ASIR in different countries and territories by SDI. **(B)** ASDR in different countries and territories by SDI. **(C)** Age-standardized DALY rate in different countries and territories by SDI.

Supplementary Figure S3 Decomposition analysis of global acute glomerulonephritis ASRs from 1990 to 2021 in males and females.
