# Supplementary figures and images for: Global, regional, and national burden of acute glomerulonephritis from 1990 to 2021 and future trend predictions until 2036: a systematic analysis using the Global Burden of Disease Study 2021
Source: Front Public Health. 2025 Jul 16;13:1593055. doi: 10.3389/fpubh.2025.1593055 (PMC12307507; doi:10.3389/fpubh.2025.1593055)

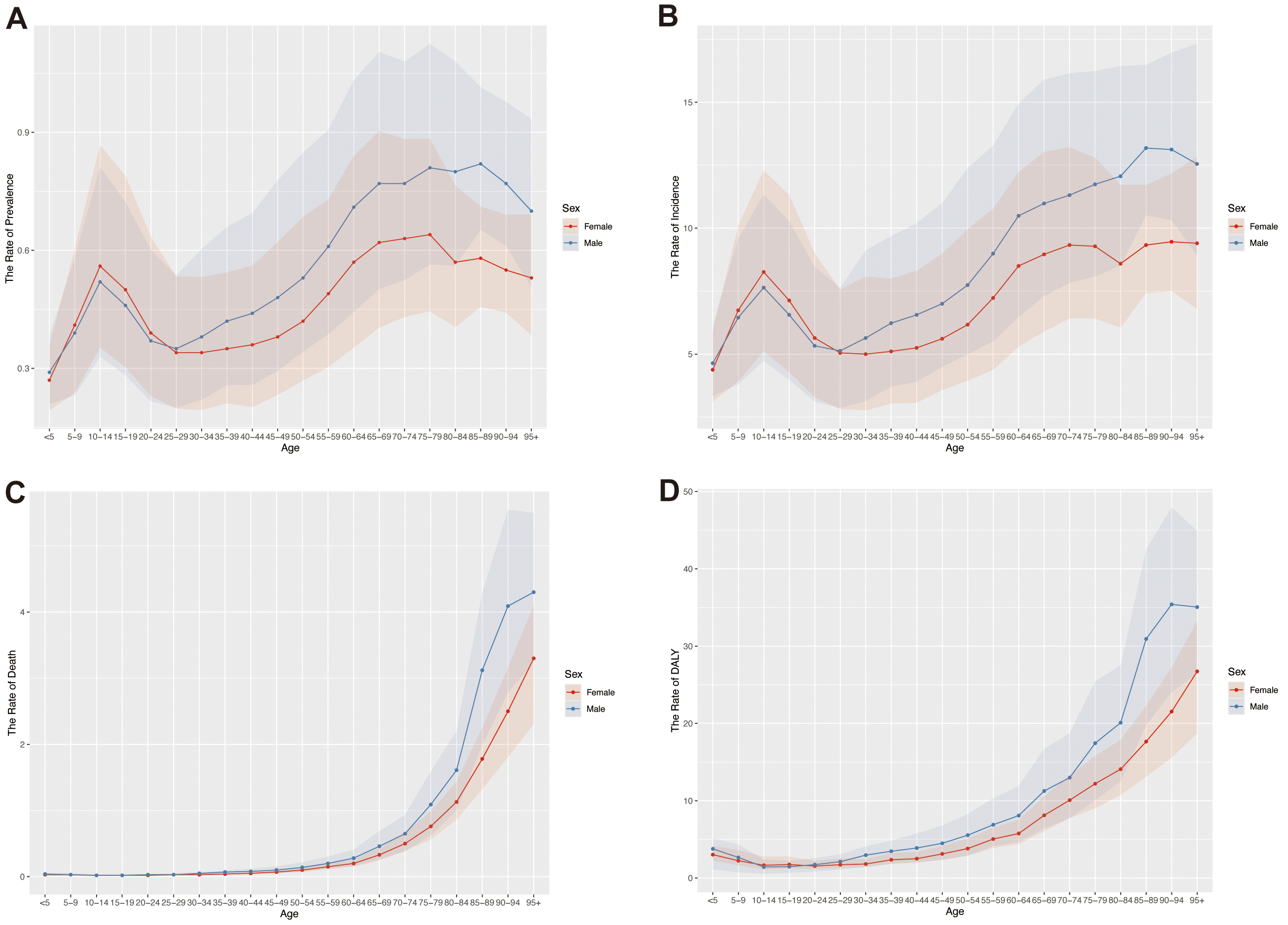

Supplement: Supplementary Figure S1 — Age-specific rate of prevalence, incidence, death, and DALY of acute glomerulonephritis by sex in 2021. (A) Rate of prevalence in different age groups. (B) Rate of incidence in different age groups. (C) The rate of death in different age groups. (D) Rate of DALY in different age groups. [file Image_1.tif]

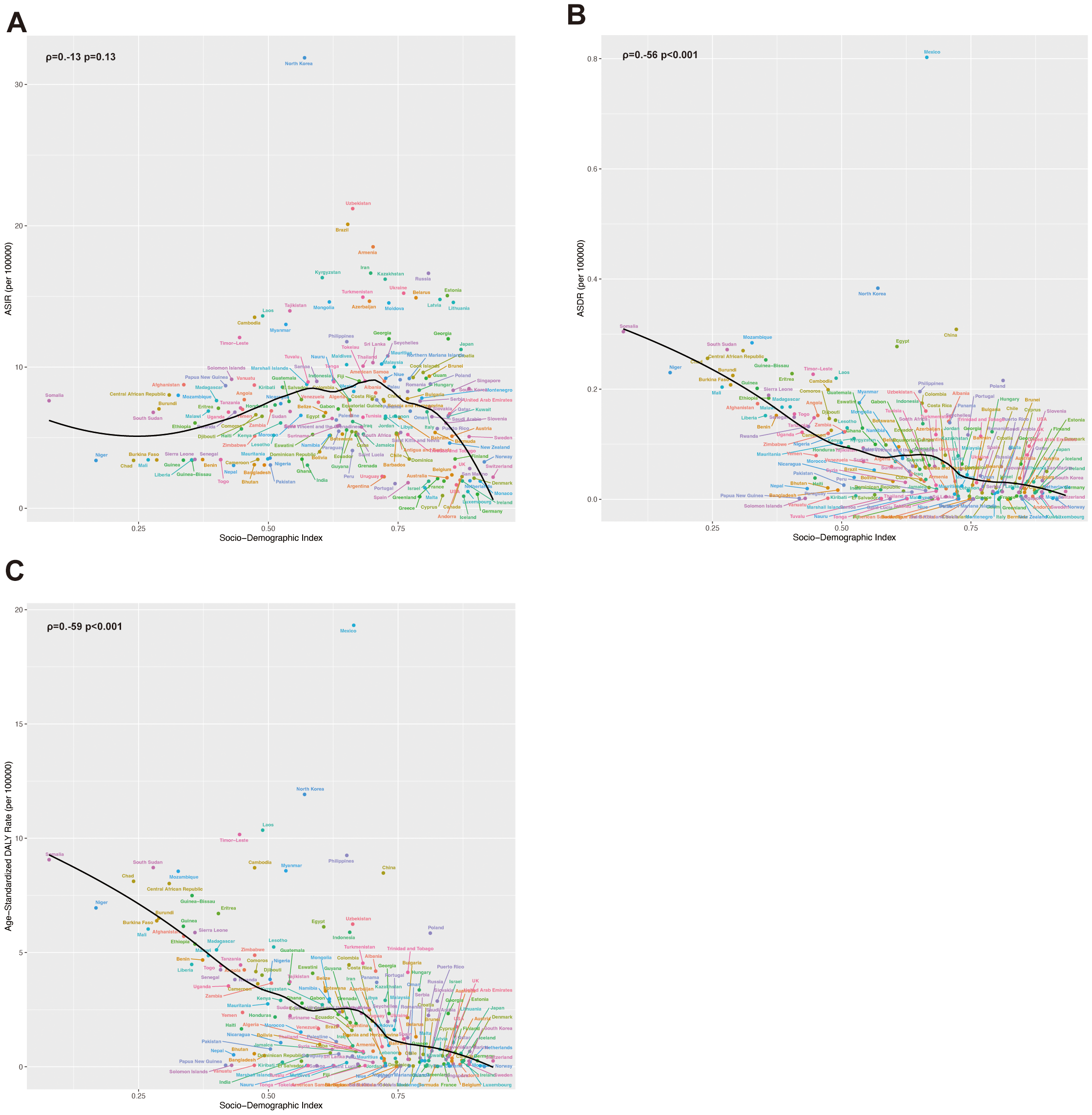

Supplement: Supplementary Figure S2 — ASRs of incidence, death, and DALY of acute glomerulonephritis and SDI from 1990 to 2021, by country and territory. (A) ASIR in different countries and territories by SDI. (B) ASDR in different countries and territories by SDI. (C) Age-standardized DALY rate in different countries and territories by SDI. [file Image_2.tif]

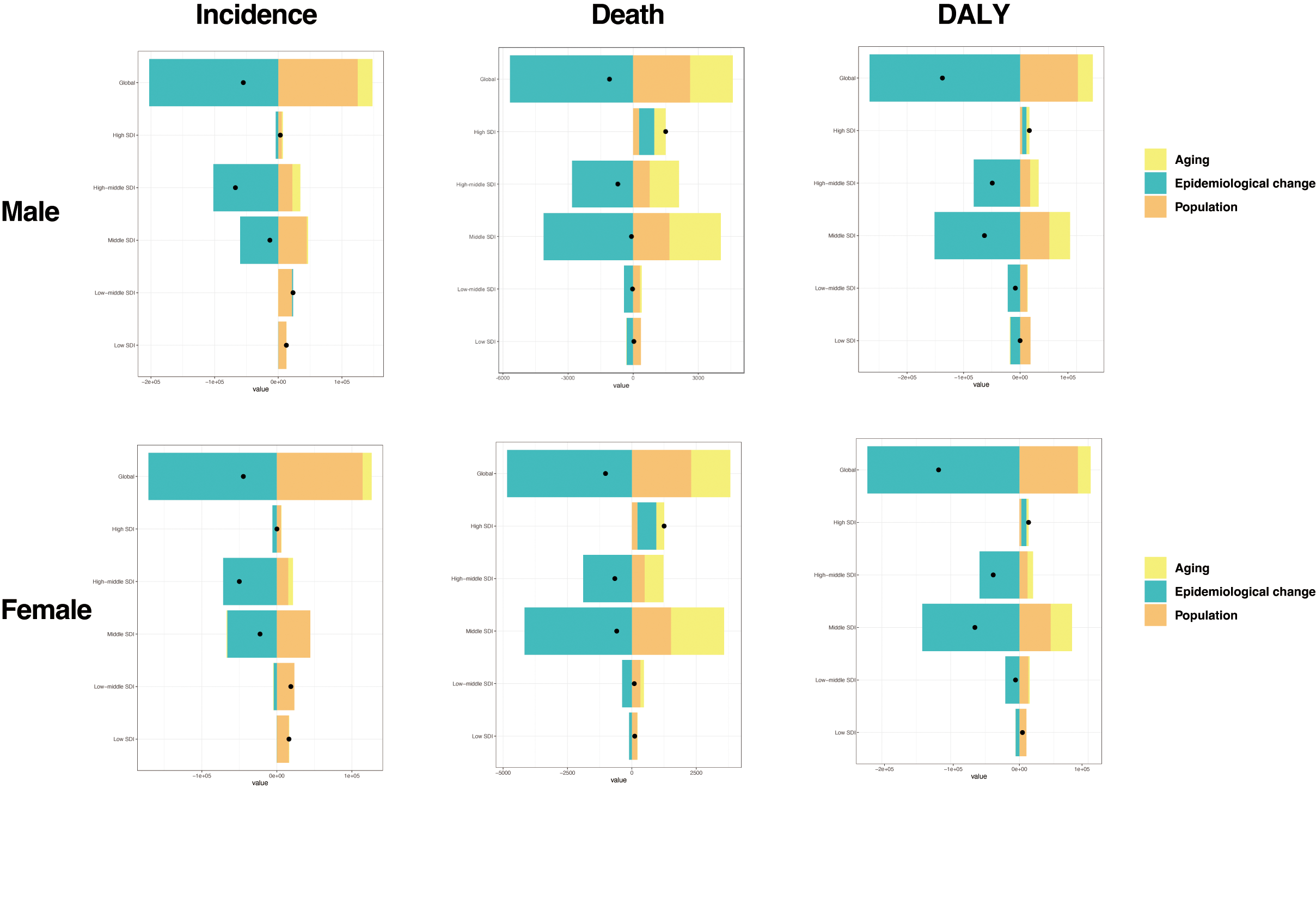

Supplement: Supplementary Figure S3 — Decomposition analysis of global acute glomerulonephritis ASRs from 1990 to 2021 in males and females. [file Image_3.tif]
