# Supplementary material for: Global, regional, and national burden of acute glomerulonephritis from 1990 to 2021 and future trend predictions until 2036: a systematic analysis using the Global Burden of Disease Study 2021
Source: Front Public Health. 2025 Jul 16;13:1593055. doi: 10.3389/fpubh.2025.1593055 (PMC12307507; doi:10.3389/fpubh.2025.1593055)
Supplement: Supplementary file 4 [file Data_Sheet_1.docx]

| **Countries and regions** | **ASIR in 2021 (95% UI)** |
| --- | --- |
| Democratic People's Republic of Korea | 31.89 (27.03,37.75) |
| Taiwan (Province of China) | 23.45 (17.72,28.29) |
| Uzbekistan | 21.21 (17.41,25.52) |
| Brazil | 20.1 (17.02,23.6) |
| Armenia | 18.51 (14.78,23.12) |
| Iran (Islamic Republic of) | 16.65 (14.63,18.81) |
| Russian Federation | 16.64 (14.16,19.33) |
| Kyrgyzstan | 16.33 (13.21,20.38) |
| Kazakhstan | 16.22 (13,20.21) |
| Ukraine | 15.23 (12.92,17.77) |

**Supplementary Table S1. Countries and regions with the highest ASIR.**

**Supplementary Table S2. Countries and regions with the lowest ASIR.**

| **Countries and regions** | **ASIR in 2021 (95% UI)** |
| --- | --- |
| Greece | 0.83 (0.61,1.04) |
| Iceland | 0.86 (0.65,1.08) |
| Cyprus | 0.9 (0.66,1.14) |
| Germany | 1 (0.75,1.24) |
| Luxembourg | 1.19 (0.89,1.5) |
| Malta | 1.46 (1.1,1.85) |
| United States of America | 1.66 (1.48,1.87) |
| Portugal | 1.75 (1.39,2.17) |
| Spain | 1.81 (1.33,2.36) |
| Israel | 1.92 (1.44,2.41) |

**Supplementary Table S3. Countries and regions with the highest ASDR.**

| **Countries and regions** | **ASDR in 2021 (95% UI)** |
| --- | --- |
| Mexico | 0.80 (0.68,0.93) |
| Democratic People's Republic of Korea | 0.38 (0.19,0.62) |
| China | 0.31 (0.21,0.40) |
| Somalia | 0.30 (0.10,0.84) |
| Mozambique | 0.28 (0.08,0.66) |
| Egypt | 0.28 (0.18,0.38) |
| South Sudan | 0.27 (0.08,0.63) |
| Central African Republic | 0.27 (0.10,0.48) |
| Chad | 0.26 (0.08,0.60) |
| Guinea-Bissau | 0.25 (0.086,0.52) |

**Supplementary Table S4. Countries and regions with the lowest ASDR.**

| **Countries and regions** | **ASDR in 2021 (95% UI)** |
| --- | --- |
| Norway | <0.0001 |
| Fiji | 0.0001 (0.0001,0.0002) |
| Barbados | 0.0001 (0,0.0001) |
| Bermuda | 0.0001 (0.0001,0.0002) |
| Guam | 0.0001 (0,0.0001) |
| Antigua and Barbuda | 0.0003 (0.0002,0.0004) |
| American Samoa | 0.0004 (0.0002,0.0006) |
| Cook Islands | 0.0004 (0.0002,0.0007) |
| Saint Kitts and Nevis | 0.0005 (0.0003,0.0007) |
| New Zealand | 0.0007 (0.0005,0.0009) |

**Supplementary Table S5. Countries and regions with the highest age-standardized DALY rate.**

| **Countries and regions** | **Age-standardized DALY rate in 2021 (95% UI)** |
| --- | --- |
| Mexico | 19.32 (16.66,22.42) |
| Democratic People's Republic of Korea | 11.92 (5.79,18.83) |
| Lao People's Democratic Republic | 10.35 (4.59,18.27) |
| Timor-Leste | 10.17 (4.98,17.86) |
| Philippines | 9.25 (6.77,12.06) |
| Somalia | 9.06 (3.19,21.19) |
| South Sudan | 8.72 (2.91,18.16) |
| Cambodia | 8.71 (3.24,15.53) |
| Myanmar | 8.58 (3.66,13.94) |
| Mozambique | 8.56 (2.50,19.25) |

**Supplementary Table S6. Countries and regions with the lowest age-standardized DALY rate.**

| **Countries and regions** | **Age-standardized DALY rate in 2021 (95% UI)** |
| --- | --- |
| Norway | 0.012 (0.007,0.018) |
| Barbados | 0.019 (0.012,0.03) |
| Bermuda | 0.023 (0.016,0.034) |
| Greenland | 0.023 (0.013,0.037) |
| Antigua and Barbuda | 0.027 (0.019,0.037) |
| Guam | 0.032 (0.02,0.051) |
| Fiji | 0.032 (0.02,0.049) |
| New Zealand | 0.035 (0.027,0.045) |
| Saint Kitts and Nevis | 0.035 (0.025,0.048) |
| American Samoa | 0.035 (0.022,0.053) |

**Supplementary Table S7. Sensitivity analysis result of decomposition analysis.** A positive overall difference indicates an increased estimate value (incidence, death or DALY) from 1990 to 2021. A negative overall difference indicates declined estimate value from 1990 to 2021. The directionality of the effect estimates remained consistent with the original analysis.

| **Both** | | | |
| --- | --- | --- | --- |
| **Location_name** | **Incidence overall difference** | **Death overall difference** | **DALY overall difference** |
| **Low SDI** | 29027.98 | 141.44 | 4369.96 |
| **Low-middle SDI** | 41443.72 | 63.57 | -14119.85 |
| **Middle SDI** | -37604.9 | -724.34 | -128502.97 |
| **High-middle SDI** | -117388.76 | -1373.65 | -86677.79 |
| **High SDI** | 3226.92 | 2668.52 | 28930.41 |
| **Global** | -99530.18 | -2115.28 | -255145.56 |
| **Male** | | | |
| **Location_name** | **Incidence overall difference** | **Death overall difference** | **DALY overall difference** |
| **Low SDI** | 12706.09 | 31.45 | -31.85 |
| **Low-middle SDI** | 23130.74 | -30.21 | -8413.36 |
| **Middle SDI** | -13187.13 | -79.4 | -63152.76 |
| **High-middle SDI** | -67315.33 | -707.92 | -49266.33 |
| **High SDI** | 3163.39 | 1497.31 | 16382.96 |
| **Global** | -54889.59 | -1092.07 | -137545.71 |
| **Female** | | | |
| **Location_name** | **Incidence overall difference** | **Death overall difference** | **DALY overall difference** |
| **Low SDI** | 16139.76 | 109.98 | 4401.81 |
| **Low-middle SDI** | 18601.03 | 93.09 | -5691.9 |
| **Middle SDI** | -22423.9 | -588.81 | -64796.41 |
| **High-middle SDI** | -50004.92 | -662.82 | -38108.15 |
| **High SDI** | 97.2 | 1251.4 | 13253.02 |
| **Global** | -44640.59 | -1023.22 | -117599.85 |
